# Building Global Perspectives in a Diverse World

**DOI:** 10.1093/geroni/igaa057.2987

**Published:** 2020-12-16

**Authors:** Allen Glicksman, Lauren Ring, Michael Liebman, Misha Rodriguez

**Affiliations:** 1 Philadelphia Corporation for Aging, Philadelphia, Pennsylvania, United States; 2 IPQ Analytics, LLC, Kennett Square, Pennsylvania, United States; 3 Asociación Puertorriqueños en Marcha, Philadelphia, Pennsylvania, United States

## Abstract

There is a growing emphasis on diversity and its impact on health care. Our research has focused on migrants who are Mandarin speaking Chinese and Puerto Rican. Through a series of focus groups conducted in their native languages we discovered that barriers to service access differ across the two groups. In addition, many of these services are administered by individual states rather than the federal government. The diversity across migrant groups and state policies creates a challenge in using findings to establish national or international standards for best practices with older migrants. How then can we apply lessons about diversity and the impact of programs on a national and international scale? We propose the use of Social Interaction Modeling as a method for understanding patterns of behavior at both national and international levels, while preserving the unique character of each migrant group and the context within which they live.

